# 2058: Are atrial fibrillation patient-reported outcomes associated with person and environment characteristics?

**DOI:** 10.1017/cts.2017.241

**Published:** 2018-05-10

**Authors:** Kelly Gleason, Cheryl Renee Dennison Himmelfarb

## Abstract

OBJECTIVES/SPECIFIC AIMS: (1) Determine person (sex, age, education level), environment (marital status, living alone, insurance), and health and illness (BMI, type of AF, comorbidities) characteristics that are associated with outcomes (QOL, symptom severity, and emotional and functional status). (2) Determine the association of symptom management strategies (ablation, cardioversion, and rate and rhythm control medications) and outcomes (QOL, symptom severity, and emotional and functional status). (3) Test person (sex, age, and education level) and environment (marital status, living alone, insurance) characteristics as moderators of the effect of symptom management strategies (ablation, cardioversion, and rate and rhythm control medications) on outcomes (QOL, symptom severity, and emotional and functional status). METHODS/STUDY POPULATION: AF patients (≥18 years of age) already enrolled in the PaTH study will be included. To date, 1026 total participants have been enrolled. Based on the enrolled participants, 92% (945) of our study population are Caucasian and 36% (362) are female. The age range of the enrolled participants is: 2% (16) 18−39, 4% (42) 40−49, 11% (108) 50−59, 33% (343) 60−69, 34% (353) 70−79, and 16% (162) 80+. Participants are recruited through in-person, email, phone, patient portal messaging and post mail techniques to ensure a representative sample. The PaTH study integrates electronic health record and insurance claims data with patient-reported outcome measures collected through online surveys. RESULTS/ANTICIPATED RESULTS: We hypothesize that sex, older age, low education level, living alone, absence of partner, absence of insurance coverage, high BMI, and a high number of comorbidities will be associated with lower QOL, high symptom severity, and low emotional and functional status. We further hypothesize that symptom management strategies will be associated with higher QOL, low symptom severity, and high emotional and functional status, and that these associations will be moderated by person and environment characteristics. DISCUSSION/SIGNIFICANCE OF IMPACT: The proposed research is an important first step in determining potential causes of person and environment differences in symptom severity. It will lead to tailored symptom management interventions for individuals most at risk for experiencing high symptom severity.

